# Complicated diaphragmatic hernia post laparoscopic Nissen fundoplication: a case report

**DOI:** 10.1186/s12245-025-01039-3

**Published:** 2025-11-13

**Authors:** Ahmed Maher, Ahmed Tayel, Karim Darwesh

**Affiliations:** Department of Pediatric Surgery, Faculty of Medicine, Alexandria University, Alexandria, Egypt

**Keywords:** Gastric volvulus, Nissen fundoplication, GERD

## Abstract

**Background:**

The definition of gastroesophageal reflux disease is gastroesophageal reflux with associated symptoms or complications. Because parents or guardians perceive the disease’s symptoms differently, it is difficult to determine how common it is. Although gastric volvulus and wrap migration are well-known complications following Nissen fundoplication, the herniation in our case was caused by a defect medial to the hiatus, which could potentially compromise the patient’s life in the future.

**Case presentation:**

We present a rare complication post laparoscopic Nissen fundoplication, four years male Egyptian child presented to emergency department by shock, rigid abdomen. X-ray and CT scan abdomen showed herniated stomach with ischemic volvulus of the stomach and pneumoperitoneum, exploration was done and case managed successfully.

**Conclusion:**

Gastric volvulus is a complication with high morbidity and mortality outcomes that could be missed or misdiagnosed, especially post laparoscopic fundoplication, high index of suspicion is the key to detect such complications. Defects in diaphragm could be present in pediatric patients because of congenital or acquired causes, careful inspection of diaphragm during laparoscopic fundoplication is an important step to detect possible defects to minimize post operative complications.

## Background

Gastroesophageal reflux disease could be defined as gastroesophageal reflux with symptoms or complication of gastroesophageal reflux, the prevalence of that disease is hard to be exactly known because of different symptoms interpretation by parents or guardians [1]. 

The condition commonly resolved by age of 2 years, however severe cases who presented by erosive gastritis, asthma and failure to thrive could need to be surgically corrected [1]. 

The commonly used technique for repairing GERD is Nissen fundoplication which could be done by laparotomy or laparoscopy [1]. 

Complication post Nissen fundoplication could be early complication or late one, injury to viscera during trocar insertion, injury to vagus nerve during dissection, tight wrap, recurrence, wrap migration or complication related to gastrostomy in cases required gastrostomy with the fundoplication [2]. 

## Case presentation

**Fig. 1:**
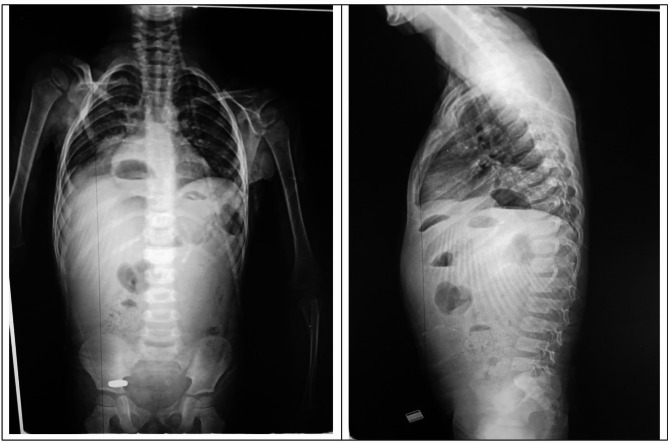
X-ray chest and abdomen

**Fig. 2:**
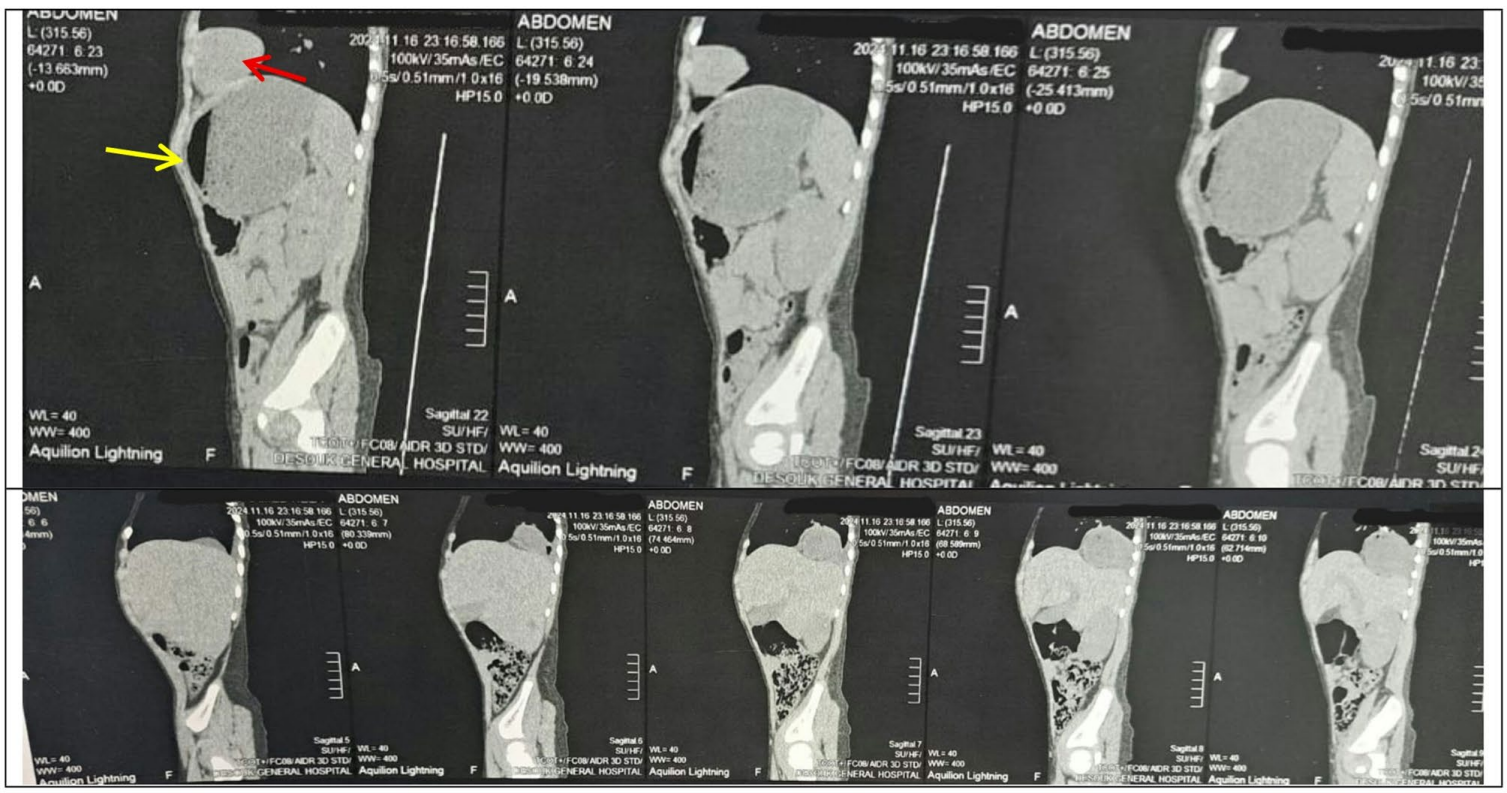
Ct chest and abdomen red arrow shows stomach in chest, yellow arrow shows pneumoperitoneum

**Fig. 3:**
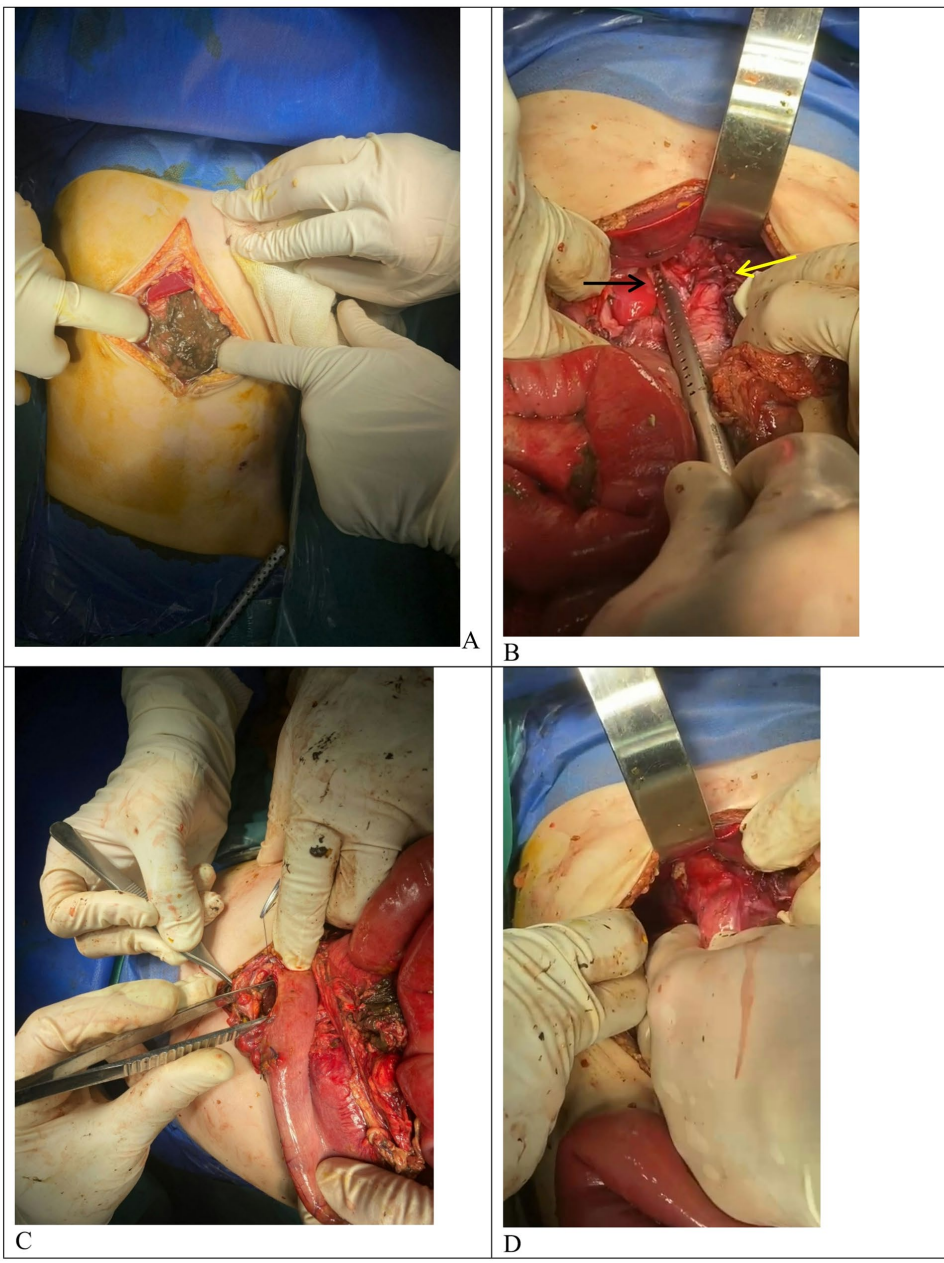
Operative view. A: upon entry of abdomen, B: defect in diaphragm with suction tube in the defect black arrow, the yellow arrow point to hiatus, C: one of the gastric perforations, D: the wrap in place without migration nor disruption

We present a rare complication post laparoscopic Nissen fundoplication, four years male Egyptian child presented to emergency department by shock, rigid abdomen. Past surgical history was laparoscopic Nissen fundoplication one year back, through that year patient was non symptomatic. Rapid resuscitation started, x-ray abdomen done (Fig. 1) showed pneumoperitoneum with bowel in chest, next step was CT scan (Fig. 2) with contrast that was done which confirmed the x-ray finding identifying stomach present in chest. Patients were stabilized, then shifted to emergency exploration.

midline exploratory incision (Fig. 3): gastric content was the initial finding upon entering the abdomen, stomach wasn’t in left hypochondrium, but it was in chest through defect in diaphragm about three centimeters medial to the esophageal hiatus. The gastroesophageal junction was in its place and the wrap was intact, the stomach was delivered down to the abdomen through that defect where there was a combined mesentero-axial and organo-axial volvulus of the stomach in addition to five perforations in the stomach: three in the anterior wall, one in the posterior wall and the last one on the greater curvature. Derotation of stomach was done, refashioning of perforations edges was done with direct closure using Vicryl^®^ 4/0 sutures, gastropexy was done at two points using Vicryl^®^ 3/0 then closure of the defect in the diaphragm using silk 3/0 sutures. The esophageal hiatus was inspected, and the wrap was intact, The rest of the bowel was healthy, irrigation of the abdomen with warm normal saline, two drains inserted then closure of abdomen in layers.

Post operative the patient was on mechanical ventilator, inotropes. 48 h later he was extubated and weaned from inotropes, feeding was started 10 days post operative gradually till he was discharged two weeks post operative. On follow up one month later, the patient is doing fine follow up CT scan showed no herniation, stomach was in place.

## Discussion

GER is a commonly seen condition in pediatrics; it can be normal until the lower esophageal sphincter matures. With maturation of the LES, these symptoms are often resolved spontaneously. Conservative management could be effective in alleviating the symptoms however fundoplication can provide a very effective surgical treatment for the long-term cure of this condition [3]. 

The first operations to treat hiatal hernia were carried out at the beginning of the twentieth century. from 1946 till 1951. Rudolph Nissen, in 1956, was the first to publish a total 360° fundic wrap around the distal esophagus, as a treatment for GERD. Many modifications followed. In 1963 Toupet’s partial fundoplication, in 1967 the posterior gastropexy (Hill) and Belsey’s operation (Belsey Mark IV), in 1968 Collis operation, later Collis-Nissen, in 1978 prosthetic repair (Angelchik), and in 1993 the first laparoscopic fundoplication in children. Nowadays, the standard surgical technique which is most widespread is the laparoscopic Nissen fundoplication. Difficult to say exactly, but we may assume that 90% of all anti-reflux procedures performed worldwide are Nissen, and the majority are done laparoscopically [4]. 

The laparoscopic Nissen fundoplication is usually done using four trocars 5 mm, one of them used as a retractor for the left lobe of the liver that is left for the assistant surgeon, great care for its tip is important as it could traumatize the diaphragm and be missed.

Gastric volvulus is the abnormal rotation of the stomach over itself. Although it is a rare complication post fundoplication, it had been described among only a few cases mainly in adult patients following laparoscopic Nissen fundoplication [5]. 

Tight wrap disrupted fundoplication and herniated or migrated wrap are well known complication post fundoplication which could present by recurrence of symptoms. The herniated wrap could be part of stomach that herniate through hiatus beside the wrap or the whole wrap migrated into chest [5]. 

In our case the herniation occurred through a defect in diaphragm medial to the hiatus, which was not documented before in literature, although the patient remains non symptomatic for twelve months post fundoplication. Herniated stomach was complicated by both mesentero-axial and organo-axial volvulus. Our message is to carefully inspect the diaphragm during fundoplication and to be careful during laparoscopic fundoplication procedure, high index of suspension of acute abdomen post laparoscopic fundoplication is the key for diagnosis.

## Conclusion

Gastric volvulus is a complication with high morbidity and mortality outcomes that could be missed or misdiagnosed, especially post laparoscopic fundoplication, high index of suspicion is the key to detect such complications. Defects in diaphragm could be present in pediatric patients because of congenital or acquired causes, careful inspection of diaphragm during laparoscopic fundoplication is an important step to detect possible defects to minimize post operative complications.

## Data Availability

No datasets were generated or analysed during the current study.

## Declarations

### Previous presentation

The study was neither presented in any meeting nor submitted for publication in other journals.

## Abbreviations

GERD: Gastroesophageal reflux disease
GER: Gastroesophageal reflux
CT scan: Computed tomography scan
